# A protocol for a systematic review for perioperative pregabalin use

**DOI:** 10.1186/2046-4053-1-40

**Published:** 2012-09-13

**Authors:** Naveen Eipe, John Penning, Mohammed Ansari, Fatemeh Yazdi, Nadera Ahmadzai

**Affiliations:** 1 The Ottawa Hospital (TOH), 1053 Carling Avenue, Suite B310, Ottawa, Ontario K1Y 4E9, Canada; 2 Knowledge Synthesis Group, Centre for Practice-Changing Research, Ottawa Hospital Research Institute, Ottawa, Canada

**Keywords:** Analgesic, Opioids, Perioperative pain, Pregabalin, Surgery

## Abstract

**Background:**

Perioperative pain management has recently been revolutionized with the recognition of novel mechanisms and introduction of newer drugs. Many randomized trials have studied the use of the gabapentinoid anti-epileptic, pregabalin, in acute pain. Published systematic reviews suggest that using pregabalin for perioperative pain management may decrease analgesic requirements and pain scores, at the expense of troublesome side effects. A major limitation of the extant reviews is the lack of rigorous investigation of clinical characteristics that would maximize the benefit harms ratio in favor of surgical patients. We posit that effects of pregabalin for perioperative pain management vary by the type of surgical pain model and propose this systematic review protocol to update previous systematic reviews and investigate the heterogeneity in findings across subgroups of surgical pain models.

**Methods/Design:**

Using a peer-reviewed search strategy, we will search key databases for clinical trials on perioperative pregabalin use in adults. The electronic searches will be supplemented by scanning the reference lists of included studies. No limits of language, country or year will be imposed. Outcomes will include pain; use of co-analgesia, particularly opioids; enhanced recovery; and drug-related harms. We will focus on the identification of surgical models and patient characteristics that have shown benefit and adverse effects from pregabalin.

Two clinical experts will independently screen the studies for inclusion using eligibility criteria established *a priori*. Data extracted by the reviewers will then be verified. Publication bias will be assessed, as will risk of bias using the Cochrane Risk of Bias tool. Meta-analysis and meta-regression are planned if the studies are deemed statistically, methodologically and clinically homogenous. Evidence will be graded for its strength for a select number of outcomes.

**Discussion:**

We will explore the findings of perioperative clinical trials studying the use of pregabalin for acute pain. We will comment on the implications of the findings and provide further direction for the appropriate use of pregabalin in acute pain. This protocol will attempt to bridge the growing gap between clinical experience and emerging evidence, and has the potential to aid future guideline development in the perioperative use of pregabalin.

**Trial registration:**

PROSPERO registration number CRD42012002078

## Introduction

Pregabalin is a well-recognized central nervous system depressant that was first introduced in 2004 as an epileptic and more potent successor to gabapentin [1]. Though gabapentin was also initially approved for treating seizures, it found widespread use in the treatment of chronic pain, and this was followed by a number of clinical trials reporting benefits with gabapentin in acute postoperative pain. Pregabalin has almost followed gabapentin on this path of clinical utilization - currently the United States Food and Drug Administration (FDA) and Health Canada approve the use of pregabalin for seizures and chronic pain (post herpetic neuralgia, fibromyalgia and diabetic peripheral neuropathy) [1]. In the European Union, pregabalin is also approved for use in generalized anxiety disorders [2]. Therefore the use of the gabapentinoids (gabapentin and pregabalin) for acute postoperative pain, though widely reported, is still off-label. The off-label use of gabapentin has been controversial and subject to a large financial settlement by industry for illegal and fraudulent promotion of unapproved uses [3].

The gabapentinoids are unique in their mechanism of action, which also explains most of their clinically relevant pharmacology. They bind to the alpha2delta subunit of the voltage-gated calcium channel in the central nervous system and this decreases the release of a variety of neurotransmitters. While their primary use continues as potent anti-epileptics and in chronic pain, their emerging role as anti-hyperalgesics has been of use in perioperative pain management. Other investigators have suggested that the anxiolytic, sedative and sleep restorative properties of these drugs may additionally benefit patients with pain.

There is well-documented evidence and extensive experience with gabapentinoids in perioperative pain management [4]. Similar clinical benefit has been reported with pregabalin; when compared with gabapentin, pregabalin has greater potency, better bioavailability and fewer side effects [5]. Clinical trials with pregabalin in perioperative pain followed the anecdotal and observational evidence that suggested its usefulness in treating acute neuropathic symptoms and indicated an apparent opioid-sparing effect [5]. As such, the off-label use of pregabalin in the perioperative period for acute pain has continued to be investigated and reported. The focus of perioperative pain management has shifted from just treating the pain to wider enhanced recovery programs (early ambulation, early feeding and shortened length of stay), fewer side effects and some long-term benefits. This wider impact of pregabalin in enhanced recovery programs and for reduced incidence of chronic postsurgical pain continues to be evaluated.

In 2009, a Cochrane systematic review evaluated the available evidence for acute and chronic pain management with pregabalin. Six perioperative clinical trials were included for analysis and these authors concluded that there is no evidence to support the use of pregabalin in acute pain [6]. Further accumulating evidence was reported in the next meta-analysis by Zhang and colleagues in 2010 that included 11 randomized controlled trials (RCTs) [7]. They reported that, though pregabalin did not reduce postoperative pain scores, it reduced opioid consumption and opioid-related adverse effects after surgery. In 2011, as perioperative clinical trials in pregabalin continued to be reported, Engelman and Cateloy published their meta-analysis of 18 RCTs on the use of pregabalin for acute pain [8]. They reported that pregabalin increased the risk of dizziness, light-headedness and visual disturbances, but decreased the occurrence of postoperative nausea and vomiting (PONV) in patients who did not receive anti-PONV prophylaxis. They concluded that, in the postoperative period, pregabalin provides additional analgesia but at the cost of additional adverse effects.

Our clinical experience with this drug has been extensive and we have previously published a clinical algorithm guiding appropriate patient and surgical procedure selection to maximize the benefit and minimize harms of perioperative pregabalin in acute pain management [9]. In our experience, patients with preoperative chronic pain, having surgery at the site of pain, or having otherwise painful surgical procedures benefit most from perioperative use of pregabalin. Relevant populations could include those undergoing spinal surgery, amputations and joint replacement, or others with higher prevalence of chronic postsurgical pain (for example, breast surgery and dental extractions). The balance of benefit to harm is likely to be unfavorable for patients undergoing procedures that are not painful or are not associated with chronic pain (for example, cholecystectomy or laparoscopic gynecological procedures). Our algorithm also points out that side effects with pregabalin (somnolence, sedation, visual disturbances, confusion and respiratory depression) may be more common in the elderly, in patients with sleep apnea and in those with renal function impairment [9].

Since the last meta-analysis by Engelman and Cateloy [8], a significant number of RCTs on the perioperative use of pregabalin for acute pain have been published. With the emergence of new evidence, and our clinical hypothesis that the balance of benefit versus harm of pregabalin use might favor only a subset of patients undergoing certain surgical procedures, a systematic review of the literature is warranted. For appropriate clinical decision-making, and possibly to provide an evidence basis for future clinical practice guidelines on the perioperative use of pregabalin for pain control, our proposed review will primarily focus on an exploration of the heterogeneity in effect estimates of benefit and harms across the clinical and methodological diversity observed in the extant literature.

### Aims and objectives

The aim of this systematic review is to evaluate the effectiveness and harms of perioperative pregabalin in the management of postoperative pain for the diverse patients undergoing various surgical procedures. To this end, the proposed systematic review will answer the following questions:

1. When compared with standard multimodal analgesia, what are the comparative effectiveness and harms of the co-administration of pregabalin in the perioperative pain management of adult patients?

2. Is there a definitive opioid-sparing advantage of pregabalin (for example, lower risk of nausea, vomiting, somnolence, opioid use, and other opioid-related side effects) when used for perioperative pain management in adults?

3. For questions 1 and 2 above, what clinical and study methodological characteristics explain the heterogeneity in results?

## Methods/Design

The conduct of our systematic review will comply with the methodological standards outlined in the *Cochrane Handbook for Systematic Reviews of Interventions*, and methods papers published for the Effective Healthcare Program of the Agency for Healthcare Research and Quality [10-12]. Reporting of our review findings will adhere to the standards for the Preferred Reporting Items for Systematic reviews and Meta-Analyses [13]. Our review has been prospectively registered (CRD42012002078) with the International Prospective Register of Systematic Reviews (PROSPERO [14]).

### Eligibility criteria

Table 1 lists our study eligibility criteria. We will include studies published in the English language; however, our search of the literature will not be restricted by language of publication. As such, we will report the number of studies excluded because of language ineligibility. Studies will not be excluded based on publication status, publication date or reported outcomes. As an adequate body of evidence from RCTs is anticipated, our review will exclude all non-randomized study designs.

**Table 1:** Review eligibility criteria

**Study characteristic**	**Inclusion criteria**	**Exclusion criteria**
Patient population	· Adults undergoing elective surgical procedures	· Non-surgical use, such as anti-epileptic, chronic pain, psychiatric conditions, generalized anxiety disorder
		· Trauma, burns without surgery
		· Other painful medical conditions, such as diabetic peripheral neuropathy, post herpetic neuralgia, fibromyalgia
Intervention*- Treatment*	· Oral pregabalin (any dose) administered before and/or after surgery with or without background multimodal analgesia	· Gabapentin
	· Studies of pregabalin in combination with another drug will also be included but excluded in sensitivity analyses	
Intervention*- Comparison*	· Placebo or any specific analgesic (including pregabalin in a different dose or gabapentin) with any route of administration, except sedatives and hypnotics (for example, acetaminophen, nonsteroidal anti-inflammatory drugs, opioids, tricyclic antidepressants)	
	· Comparators may or may not have been added on to background multimodal analgesia.	
Study design	· Randomized controlled trials	· Case studies
		· Observational studies
		· Retrospective study
		· Non-randomized
		· Non controlled
		· Expert opinion
Setting	· Inpatient with or without extension to outpatient settings	· Exclusively outpatient
Timing	· 48 to 24 hours before surgery to 7 days (for acute pain) and up to 6 months postsurgery (for chronic postoperative pain)	

### Information sources and literature search

Electronic search strategies will be developed and tested by an experienced medical information specialist in consultation with the Evidence based Practice Center (EPC) team. The search strategy will be peer-reviewed according to the peer review of electronic search strategies guideline [15]. Published literature will be identified through searching MEDLINE, EMBASE (via OVID) and the Cochrane Central Register of Controlled Trials database (via OVID). The strategy for MEDLINE is included as Additional file 1: Appendix 1 and will be translated as appropriate for the other databases. The search strategies will combine the use of controlled vocabulary and keywords. There will be no language or date restrictions. Animal studies will be excluded. The study design filter will be used to identify RCTs. For MEDLINE, the Cochrane Highly Sensitive Search Strategy for identifying randomized trials in MEDLINE sensitivity-maximizing version (2008 revision) and the OVID format will be used.

Additional references will be sought through scanning the reference list of systematic reviews on pregabalin for acute pain in the perioperative setting.

#### Study selection process

Two reviewers will independently screen all identified items at two levels. Level 1 screening will entail a broad screen based on item titles and/or abstracts, as available. The full-text of all items passing Level 1 screening will be retrieved for Level 2 screening: an ascertainment of final eligibility for the review. Discrepancies will be resolved by consensus or by involving a third team member. All screening will be conducted in DistillerSR (Evidence Partners Incorporated, Ottawa, Canada) using inclusion criteria developed *a priori* as described above.

### Data items and data collection process

Standardized data extraction forms, with accompanying data extraction guidelines, will be iteratively developed and pilot tested until an appropriate level of consensus is reached. Data will be extracted by one reviewer and verified by another. Errors in data abstraction will be corrected after consultation with the primary data extractor, as required. Authors of studies may be contacted for missing data or data clarification. Multiple publications of the same study will be identified and used for all relevant reported data. The multiple publications then will be linked together as companion reports, but true duplicates will be excluded.

For all studies, the following data will be extracted:

Study characteristics: first author; year of publication; country of origin; funding source; study design and setting; duration of follow-up; number randomized; number analyzed for each outcome; and number of drop-outs with reason.

Population characteristics: inclusion and exclusion criteria; patient characteristics (for example, age, gender, race) underlying disease or condition; co-morbidities (for example, kidney disease, sleep apnea); type and duration of elective surgery; anesthesia protocol (general, spinal or regional); anatomical site of surgery; pre-existing pain at the site of surgery; history of use of gabapentinoids in the last 6 months; history of past surgery at same anatomical site or a different site of surgery; and history of opioid abuse and dependence.

Intervention characteristics: dose, frequency, timing and duration of treatment; drug compliance; co-interventions (co-analgesia with dose, frequency and duration); and concomitant medications (dose, frequency and duration).

Comparison intervention characteristics: intervention description, dose, duration of treatment.

Outcomes: In addition to quantitative data for each of the following outcomes (Table 2), details of their definitions, cut-offs used for categorizations, and tools of measurements will also be extracted. Where possible, data will be extracted as number of patients with an event (as opposed to number of events) to ensure only one event is recorded per patient in cases of multiple events experienced by the same patient.

**Table 2:** Review outcomes

**Type of outcome**	**Outcome**
*Primary perioperative efficacy outcomes*	· Pain relief at rest and on movement (any measure)
	· Reduction in postoperative opioid and other analgesic consumption (dose, frequency, and time of first use of rescue analgesia)
*Secondary perioperative and long-term outcomes*	· Postoperative nausea and vomiting
	· Anxiolysis
	· Sleep restoration
	· Early ambulation
	· Early feeding
	· Length of hospital stay
	· Chronic postoperative pain
*Perioperative harms outcomes*	· Somnolence
	· Sedation
	· Visual disturbances
	· Confusion
	· Respiratory depression
	· Cognitive dysfunction
	· Withdrawal due to adverse events
	· Participants with at least one serious adverse event (as defined by the FDA)
	· Mortality
	· Participants with at least one adverse event
	· Withdrawal due to lack of pregabalin efficacy
FDA: Food and Drugs Administration	

### Risk of bias appraisal

We will use the Cochrane Risk of Bias tool in parallel to the Jadad tool to evaluate the internal validity of the design and conduct of included studies [16]. Blinding of participants, healthcare providers, and outcome assessors will be judged as low risk for objective outcomes such as mortality.

For harms outcomes, additional criteria will include precise definitions, mode of data collection (active evaluation versus passive reporting), and type of harms analysis.

For the gradable outcomes (see below), we will evaluate the risk of publication bias for the body of evidence when all of the following criteria are met:

≥ 10 studies contributing data for an outcome

studies of unequal sizes

no substantial clinical and methodological differences between smaller and larger studies

quantitative results accompanied with measures of dispersion.

### Data synthesis

Our primary outcomes are pain relief at rest and on movement (any measure) and reduction in postoperative opioid and other analgesic consumption. Other outcomes of interest are pre-specified above. We will first report our results descriptively and narratively.

Because exploration of subgroup differences in the effects of pregabalin is of main import, main analyses will be directed towards the following surgical pain models: surgery associated with severe neuropathic pain (for example, spine surgeries, joint replacement and amputations); surgery associated with chronic postsurgical pain (for example, breast surgery, hernia repair and spine surgeries); and unclassified.

We will also attempt to investigate an overall relative drug effect for outcomes across all patient populations, co-analgesia, and types of surgical models when there is no major inconsistency in effect estimates or when heterogeneity across studies cannot be explained by clinical and methodological factors. As a rule, heterogeneity that can be explained by differences amongst the studies will preclude any planned meta-analyses. All meta-analyses will be based on the DerSimonian’s and Laird’s random effects approach [17]. Statistical heterogeneity between studies will be quantified with I-squared statistics and the *P* value from the chi-squared test (*P* ≤ 0.10 instead of ≤ 0.05 will be used to determine statistical significance). Sparse data will not be meta-analyzed but described narratively.

It is anticipated that outcomes may be reported as dichotomous, continuous or count data. Relative risk for dichotomous outcomes, mean difference or standardized mean differences for continuous, and rate ratios for count data will be preferred measures of analysis. Studies with zero events in one arm will be meta-analyzed without continuity correction with either the Peto method or the Mantel-Haenszel method [11,17]. Studies with zero events in both arms will be excluded from meta-analyses.

Meta-regression with multiple study level covariates will be attempted when there are at least 10 studies in a meta-analysis. Otherwise, clinical and methodological diversity in studies will be explored in subgroup analysis for the following study level covariates, data permitting:

**Methodological covariates** - study risk of bias and short- and longer-term perioperative period.

**Clinical covariates** - surgery at the site of chronic pain and surgery away from the site of chronic pain; patients with opioid dependence or tolerance, anxiety, sleep disruption, and catastrophizing; patients with preoperative pain and patients without preoperative pain; single preoperative dose of pregabalin, multiple doses of pregabalin, and various doses of pregabalin (low dose < 150 mg/day, intermediate dose 150 to 300 mg/day and high dose > 300 mg/day); various types of surgeries; studies on patients likely to experience chronic postoperative pain; and subtypes of anesthesia protocols (general, spinal or regional).

### Grading the strength of evidence and assessment of applicability

For a given outcome, reviewers’ confidence on the body of evidence in support of a conclusion will be graded as per previous guidance [12]. Mandatory domains that will be assessed will include Risk of Bias, Consistency, Directness and Precision. Our pre-specified gradable outcomes are outcomes most likely to influence decision-making. They include: mortality, serious adverse events, respiratory depression or arrest, visual disturbances, pain relief or scores, analgesic consumption, sleep restoration, and enhanced recovery (early feeding and ambulation, and length of hospital stay). For the body of evidence, we will summarize the population, intervention, comparator, setting and study duration data that may be used to assess external validity of evidence by various stakeholders and decision-makers.

## Discussion

The gabapentinoids, pregabalin and gabapentin, are novel drugs that have probably revolutionized the management of pain [1]. Both drugs share similar anti-hyperalgesic, sedative and anxiolytic properties and are currently approved for use in chronic pain. Since its introduction into clinical practice in 2004, pregabalin has demonstrated the more favorable pharmacokinetic profile, especially a more predictable dose-independent absorption. This property and its improved side-effect profile make pregabalin highly desirable for the management of perioperative pain.

It is important to appreciate this evolution in the clinical use of the gabapentinoids, as other systematic reviews have provided combined analyses of gabapentin and pregabalin for either or both chronic and acute pain [5,18,19]. Our systematic review will focus on the use of pregabalin for acute pain.

The first narrative review published by Gajraj in December 2007 on the use of pregabalin suggested that it would be useful for acute pain and predicted that it would likely become well studied as part of multimodal analgesia [1]. The Cochrane review in 2009, however, suggested that there was no benefit in acute pain from pregabalin, which was not surprising because the evidence available was limited [6]. As more clinical trials were performed and their findings became available, systematic reviews reported that pregabalin decreased pain scores, analgesic consumption and side effects [7,8]. There was also suggestion that some indirect perioperative benefit from pregabalin resulted in decreasing analgesic (opioid) consumption, thereby decreasing nausea and vomiting, the well-known side effects of opioids [8]. The limitations of the previous meta-analysis include the wide variability of the studies, the pregabalin dose and regimes, and the anesthetic and analgesic protocols, as well as an inability to pool data due to heterogeneity [7].

We determined that the findings of these systematic reviews are not consistent across all patients or all surgical procedures. We have previously identified patient characteristics and surgical procedures that may benefit from pregabalin [9]. Through this systematic review, we would like to carefully explore the findings of the more recent perioperative clinical trials that have studied the use of pregabalin for acute pain. We will comment on the implications of the findings and provide further direction for the appropriate use of pregabalin in acute pain. This protocol will attempt to bridge the growing gap between clinical practice and emerging evidence. Finally, this systematic review has the potential to aid future guideline development in the perioperative use of pregabalin.

## Competing interests

JP has received honoraria from Pfizer as speaker fees for talks on acute pain. NE, MA, FY and NA declare that they do not have any competing interests.

## Authors’ contributions

NE, JP and MA were involved in the conception and design of the review protocol and approved the final version. They will also be involved in data interpretation, constructing the review, and critical revisions for important intellectual and clinical content. FY and NA reviewed and provided assistance with the content and design of the protocol and approved the final version. FY and NA will design and implement the data extractions and the risk of bias assessment of included studies; process and prepare the data for analyses; analyze, organize and interpret data; and prepare the structure of the first draft of the review. All authors read and approved the final version of this manuscript.

## Supplementary Material

Additional file 1 **Appendix 1:** Search Strategy for Pregabalin Protocol. Click here for file
