# Supplementary material for: A protocol for a systematic review for perioperative pregabalin use
Source: Syst Rev. 2012 Sep 13;1:40. doi: 10.1186/2046-4053-1-40 (PMC3472239; doi:10.1186/2046-4053-1-40)
Supplement: Additional file 1 — Appendix 1: Search Strategy for Pregabalin Protocol. [file 2046-4053-1-40-S1.doc]

**Appendix 1: Search Strategy for Pregabalin Protocol**

**Database: Ovid MEDLINE(R) In-Process & Other Non-Indexed Citations and Ovid MEDLINE(R) <1946 to Present> Search Strategy:**

1     (Lyrica or pregabalin).mp. (1232)

2     Acute pain/ or (acute adj5 pain$).tw. or severe pain$.tw. (23515)

3     exp Pain, Postoperative/ or postoperat$ pain.tw. or post-operat$ pain.tw. or post operat$ pain.tw. or post-surg$

pain.tw. or post surg$ pain.tw. (30810)

4     exp Analgesia/ or analges$.tw. (92151)

5     exp Neuralgia/ or neuralgi$.tw. or sciatica.tw. (18665)

6     Colic/ or colic$.tw. (11022)

7     Headache/ or headache$.tw. (58860)

8     Toothache/ or toothache$.tw. or dental pain$.tw. (3075)

9     Earache/ or earache$.tw. (759)

10     Dysmenorrhea/ or dysmenorrhea.tw. or dysmenorrhoea.tw. (4615)

11     exp Arthralgia/ or arthralgi$.tw. (10570)

12     neuropath$.tw. (79920)

13     or/2-12 (296914)

14     1 and 13 (592)

15     randomized controlled trial.pt. (326996)

16     controlled clinical trial.pt. (84070)

17     randomized.ab. or randomized.ti. (259378)

18     placebo.ab. (135881)

19     drug therapy.fs. (1528975)

20     randomly.ab. (178142)

21     trial.ab. (251344)

22     groups.ab. (1163398)

23     or/15-22 (2932316)

24     exp animals/ not humans.sh. (3712811)

25     23 not 24 (2503889)

26     14 and 25 (437)

27     2012$.ed. (295437)

28     26 and 27 (21)

**Database: Embase Classic+Embase <1947 to 2012 May 15> Search Strategy:**

1     Pregabalin/ (5209)

2     (pregabalin or lyrica).tw. (2407)

3     1 or 2 (5372)

4     ((acute adj5 pain$) or severe pain$).tw. (33976)

5     Postoperative pain/ or postoperat$ pain.tw. or post-operat$ pain.tw. or post operat$ pain.tw. or post-surg$ pain.tw. or post surg$ pain.tw. (41600)

6     exp *Analgesia/ or analges$.tw. (129340)

7     exp *Neuralgia/ or neuralgi$.tw. or sciatica.tw. or earache$.tw. (47973)

8     *Colic/ or colic$.tw. (15260)

9     *Headache/ or headache$.tw. (88374)

10     *Tooth pain/ or tooth pain$.tw. or toothache$.tw. or dental pain$.tw. (3288)

11     *Dysmenorrhea/ or dysmenorrhea.tw. or dysmenorrhoea.tw. (6219)

12     *Arthralgia/ or arthralgi$.tw. (10947)

13     neuropath$.tw. (109389)

14     or/4-13 (431152)

15     3 and 14 (2367)

16     Clinical trial/ (873855)

17     randomized controlled trial/ (323862)

18     Randomization/ (58313)

19     Single blind procedure/ (15872)

20     Double blind procedure/ (113374)

21     Crossover procedure/ (34098)

22     Placebo/ (214796)

23     Randomi?ed controlled trial$.tw. (74465)

24     Rct.tw. (9263)

25     Random allocation.tw. (1214)

26     Allocated randomly.tw. (1832)

27     Randomly allocated.tw. (17380)

28     (allocated adj2 random).tw. (862)

29     Single blind$.tw. (12464)

30     Double blind$.tw. (137246)

31     ((treble or triple) adj blind$).tw. (310)

32     Placebo$.tw. (183530)

33     Prospective study/ (204369)

34     or/16-33 (1275388)

35     Case study/ (24768)

36     Case report.tw. (249866)

37     Conference abstract.pt. (713089)

38     Abstract report/ or letter/ (849782)

39     or/35-38 (1816470)

40     34 not 39 (1186278)

41     15 and 40 (872)

42     2012$.em. (459540)

43     41 and 42 (43)

**Cochrane Central Register of Controlled Trials (Central) -** **Issue 5 of 12, May 2012**

| #1 | [(Pregabalin) or (Lyrica)](https://mytoh.ottawahospital.on.ca/exchweb/bin/redir.asp?URL=http://onlinelibrary.wiley.com/o/cochrane/searchHistory?mode=runquery%26qnum=1) |
| --- | --- |
| #2 | [(acute pain* ) or (severe pain*)](https://mytoh.ottawahospital.on.ca/exchweb/bin/redir.asp?URL=http://onlinelibrary.wiley.com/o/cochrane/searchHistory?mode=runquery%26qnum=2) |
| #3 | [MeSH descriptor Pain, Postoperative explode all trees](https://mytoh.ottawahospital.on.ca/exchweb/bin/redir.asp?URL=http://onlinelibrary.wiley.com/o/cochrane/searchHistory?mode=runquery%26qnum=3) |
| #4 | [MeSH descriptor Analgesia explode all trees](https://mytoh.ottawahospital.on.ca/exchweb/bin/redir.asp?URL=http://onlinelibrary.wiley.com/o/cochrane/searchHistory?mode=runquery%26qnum=4) |
| #5 | [MeSH descriptor Neuralgia explode all trees](https://mytoh.ottawahospital.on.ca/exchweb/bin/redir.asp?URL=http://onlinelibrary.wiley.com/o/cochrane/searchHistory?mode=runquery%26qnum=5) |
| #6 | [MeSH descriptor Colic explode all trees](https://mytoh.ottawahospital.on.ca/exchweb/bin/redir.asp?URL=http://onlinelibrary.wiley.com/o/cochrane/searchHistory?mode=runquery%26qnum=6) |
| #7 | [MeSH descriptor Headache explode all trees](https://mytoh.ottawahospital.on.ca/exchweb/bin/redir.asp?URL=http://onlinelibrary.wiley.com/o/cochrane/searchHistory?mode=runquery%26qnum=7) |
| #8 | [MeSH descriptor Toothache explode all trees](https://mytoh.ottawahospital.on.ca/exchweb/bin/redir.asp?URL=http://onlinelibrary.wiley.com/o/cochrane/searchHistory?mode=runquery%26qnum=8) |
| #9 | [MeSH descriptor Earache explode all trees](https://mytoh.ottawahospital.on.ca/exchweb/bin/redir.asp?URL=http://onlinelibrary.wiley.com/o/cochrane/searchHistory?mode=runquery%26qnum=9) |
| #10 | [MeSH descriptor Dysmenorrhea explode all trees](https://mytoh.ottawahospital.on.ca/exchweb/bin/redir.asp?URL=http://onlinelibrary.wiley.com/o/cochrane/searchHistory?mode=runquery%26qnum=10) |
| #11 | [MeSH descriptor Arthralgia explode all trees](https://mytoh.ottawahospital.on.ca/exchweb/bin/redir.asp?URL=http://onlinelibrary.wiley.com/o/cochrane/searchHistory?mode=runquery%26qnum=11) |
| #12 | [(postoperat* pain) or (post-operat* pain) or (post operat* pain) or (post-surg* pain) or (post surg* pain) or (analges*) or (neuralgi*) or (sciatica) or (colic*) or (headache*) or (toothache) or (dental pain) or (earache*) or (dysmenorrhea) or (dysmenorrhoea) or (arthralgi*) or (neuropath*)](https://mytoh.ottawahospital.on.ca/exchweb/bin/redir.asp?URL=http://onlinelibrary.wiley.com/o/cochrane/searchHistory?mode=runquery%26qnum=12) |
| #13 | [(#2 OR #3 OR #4 OR #5 OR #6 OR #7 OR #8 OR #9 OR #10 OR #11 OR #12)](https://mytoh.ottawahospital.on.ca/exchweb/bin/redir.asp?URL=http://onlinelibrary.wiley.com/o/cochrane/searchHistory?mode=runquery%26qnum=13) |
| #14 | [(#1 AND #13)](https://mytoh.ottawahospital.on.ca/exchweb/bin/redir.asp?URL=http://onlinelibrary.wiley.com/o/cochrane/searchHistory?mode=runquery%26qnum=14) |
| #15 | [(#14) with New in Record Status](https://mytoh.ottawahospital.on.ca/exchweb/bin/redir.asp?URL=http://onlinelibrary.wiley.com/o/cochrane/searchHistory?mode=runquery%26qnum=15) |
|  |  |
